# Clinicopathological Features of Primary Neuroendocrine Tumors of Gastrointestinal/Pancreatobiliary Tract With Emphasis on High-Grade (Grade 3) Well-Differentiated Neuroendocrine Tumors

**DOI:** 10.7759/cureus.12640

**Published:** 2021-01-11

**Authors:** Atif A Hashmi, Javaria Ali, Syed Rafay Yaqeen, Omer Ahmed, Ishaq Azeem Asghar, Muhammad Irfan, Muhammad Ghani Asif, Muhammad M. Edhi, Shumaila Hashmi

**Affiliations:** 1 Pathology, Liaquat National Hospital and Medical College, Karachi, PAK; 2 Internal Medicine, Baqai Medical University, Karachi, PAK; 3 Internal Medicine, Liaquat National Hospital and Medical College, Karachi, PAK; 4 Pathology, Ascension St. John Hospital, Detroit, USA; 5 Statistics, Liaquat National Hospital and Medical College, Karachi, PAK; 6 Pathology, Multan Medical and Dental College, Multan, PAK; 7 Neuroscience/Neurosurgery, Rhode Island Hospital, Warren Alpert Medical School of Brown University, Providence, USA; 8 Pathology, Combined Military Hospital Multan Institute of Medical Sciences, Multan, PAK

**Keywords:** neuroendocrine neoplasms, poorly differentiated neuroendocrine carcinoma (pdnec), high-grade neuroendocrine tumor, gastrointestinal tract, pancreatobiliary tract, well-differentiated neuroendocrine tumor (wdnet), neuroendocrine tumor (net)

## Abstract

Introduction

The two broad subcategories of neuroendocrine neoplasms (NENs) are well-differentiated neuroendocrine tumors (WDNETs) and poorly differentiated neuroendocrine carcinomas (PDNECs), based on tumor architecture and cytology. Grade 3 WDNETs are a subset of WDNETs that not only are high grade by mitotic activity or proliferative index but exhibit a well-differentiated histology. In this study, we evaluated the clinicopathological features of primary neuroendocrine tumors of the gastrointestinal (GI)/pancreatobiliary tract with emphasis on high-grade WDNETs, as it is a newly defined entity.

Methods

We conducted a retrospective observational study, including a total number of 122 cases of primary GI and pancreatobiliary tract NENs. Slides and blocks of all cases were retrieved from the departmental archives. Immunohistochemical stains including Ki67 were applied to selected tissue blocks of all cases. Tumors were then evaluated for their histological differentiation and tumor grade.

Results

Our results showed that the mean age of patients was 46.8 ± 17.1 years. Majority of the NENs were GI tract origin (86.9%). The most common site of tumor in gastroenteropancreatic tract was the small bowel (31.1%), followed by the stomach (26.2%). Ninety five percent of the tumors were WDNETs, of which the most common grade was G2. The mean Ki67 index was 15.8 ± 23.8. Grade 3 WDNETs were noted to have an older mean age than grades 1 and 2 WDNETs. Ten out of 102 (9.8%) WDNETs of GI tract were grade 3, compared with four out of 14 (28.6%) of pancreatobiliary tract.

Conclusion

In this study, we found that high-grade (grade 3) WDNETs were more frequent in pancreatobiliary tract than GI tract. Moreover, high-grade WDNETs were associated with a higher mean age than low-grade (grade 1-2) WDNETs. It is extremely important to recognize this subset (high grade) of WDNETs and to distinguish it from PDNECs, as the latter are known to be associated with a worse overall survival. Despite high mitotic rate/proliferative index, high-grade WDNETs are characterized by organoid architecture and monomorphic cell population.

## Introduction

Neuroendocrine neoplasms (NENs) are indolent tumors that can arise in almost all organ systems of the body and encompasses a diverse range of clinical, morphological, and genomic features with varied outcomes. They tend to arise from neuroendocrine cells, i.e., cells having characteristics of both nerve cell (ability to receive signals from the nervous system) and endocrine cells (ability to secrete hormones and peptides) [1]. Histologically, NENs are subclassified into low-proliferating well-differentiated neuroendocrine tumors (WDNETs) and high-proliferating poorly differentiated neuroendocrine carcinomas (PDNECs) [2].

The large magnitude of the gasteoenteropancreatic tract puts it as the most common site of origin for NENs, followed by lungs [3]. They constitute 2% of the malignant tumors of the gastroenteropancreatic tract [4]; however, due to the endoscopic screening and early detection, their incidence has increased in the past decades [5].

According to the fifth edition of the World Health Organization (WHO) Classification of Digestive Tumors, NENs are classified into WDNETs grade G1, G2, and G3 as well as neuroendocrine carcinomas (NECs), which constitute the poorly differentiated tumors. NECs are further subclassified into small-cell neuroendocrine carcinomas (SCNECs), large-cell neuroendocrine carcinomas (LCNECs), and finally the mixed neuroendocrine-non-neuroendocrine neoplasms (MiNENs), which are mixed epithelial neoplasms in which a neuroendocrine component is combined with a non-neuroendocrine component. This classification is based on tumor grading done by virtue of the mitotic activity and the Ki67 proliferative index [3].

The term well-differentiated, in the context of NENs, denotes the strong resemblance of tumor cells to the non-neoplastic neuroendocrine cells by virtue of their immunoexpression of general neuroendocrine markers (chromogranin A and synaptophysin) and peptide hormone production.

Grade 3 WDNETs are a subset of WDNETs, which are high grade by mitotic rate or proliferative index and exhibit a well-differentiated histology. They have a prognosis that is somewhere between the Grade 2 WDNETs and PDNECs, former being more indolent. In this study, we evaluated the clinicopathological features of primary neuroendocrine tumors of the gastrointestinal (GI)/pancreatobiliary tract with emphasis on high-grade WDNETs, as it is a newly defined entity.

## Materials and methods

We conducted a retrospective observational study at the Department of Histopathology, Liaquat National Hospital and Medical College, Karachi. A total number of 122 cases of primary GI and pancreatobiliary NENs were retrieved from the departmental archives from January 2011 until July 2020. Cases with prior history to chemoradiation were excluded from the study along with cases of metastatic neuroendocrine tumors. The specimens were received in histology lab. After the gross examination, small biopsies were submitted entirely. Resection specimens were grossed according to standard protocols as per College of American Pathologists (CAP) guidelines. After submission of resection margins, representative sections were submitted from the tumor (one section per centimeter of tumor). Tumors smaller than 2 cm were submitted entirely. Immunohistochemical stains, pan-cytokeratin, synaptophysin, chromogranin A, and Ki67, were applied to selected paraffin-embedded blocks of all cases. Slides of all cases were retrieved and reexamined for tumor grade and differentiated by experienced histopathologists. New sections were performed from tissue blocks where necessary.

Morphologically WDNETs displayed characteristic organoid architectural patterns, including nests, cords, ribbons, and rosette formation. Cytologically, these tumors displayed a monomorphic population of cells with round to oval nuclei, coarse or stippled (salt-and-pepper-like) chromatin with a granular cytoplasm.

**Figure 1:**
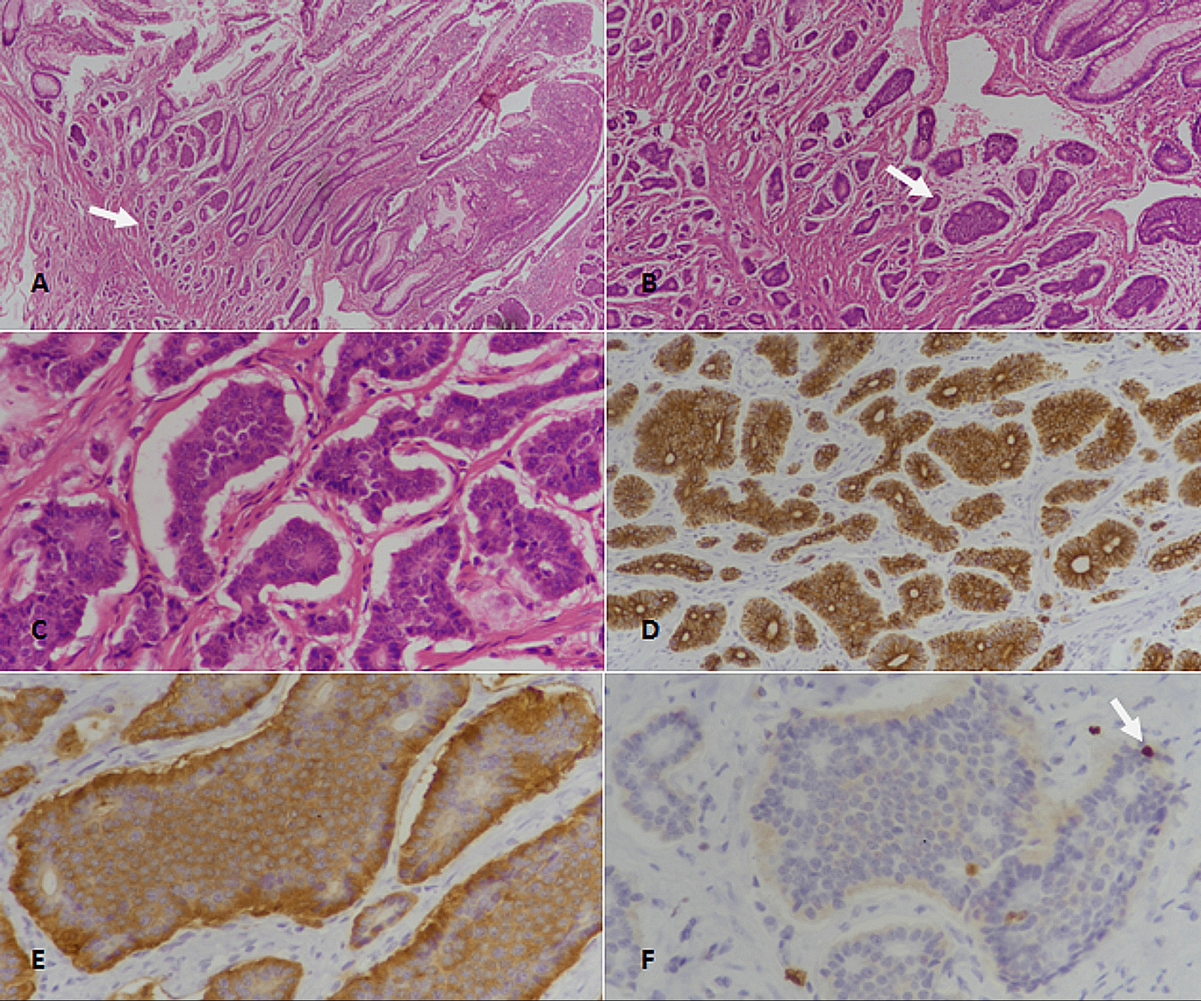
Well-differentiated neuroendocrine tumor (WDNET), grade 1 of the small intestine. (A): H&E-stained section at 40x magnification showing nests and clusters of tumor cells (arrow) in mucosa and submucosa. (B): H&E-stained section at 100x magnification revealing neuroendocrine tumor with nested architecture (arrow). (C): 400x magnification (H&E-staining) showing tumor cells with mild nuclear atypia and stippled coarse chromatin. (D): Pancytokeratin (CKAE1/AE3)-staining showing strong diffuse positivity. (E): Synaptophysin immunostaining depicting diffuse positivity. (F): Ki67 immunostaining showing less than 2% proliferative index; occasional cells show nuclear positivity (arrow). H&E: Hematoxylin and eosin.

**Figure 2:**
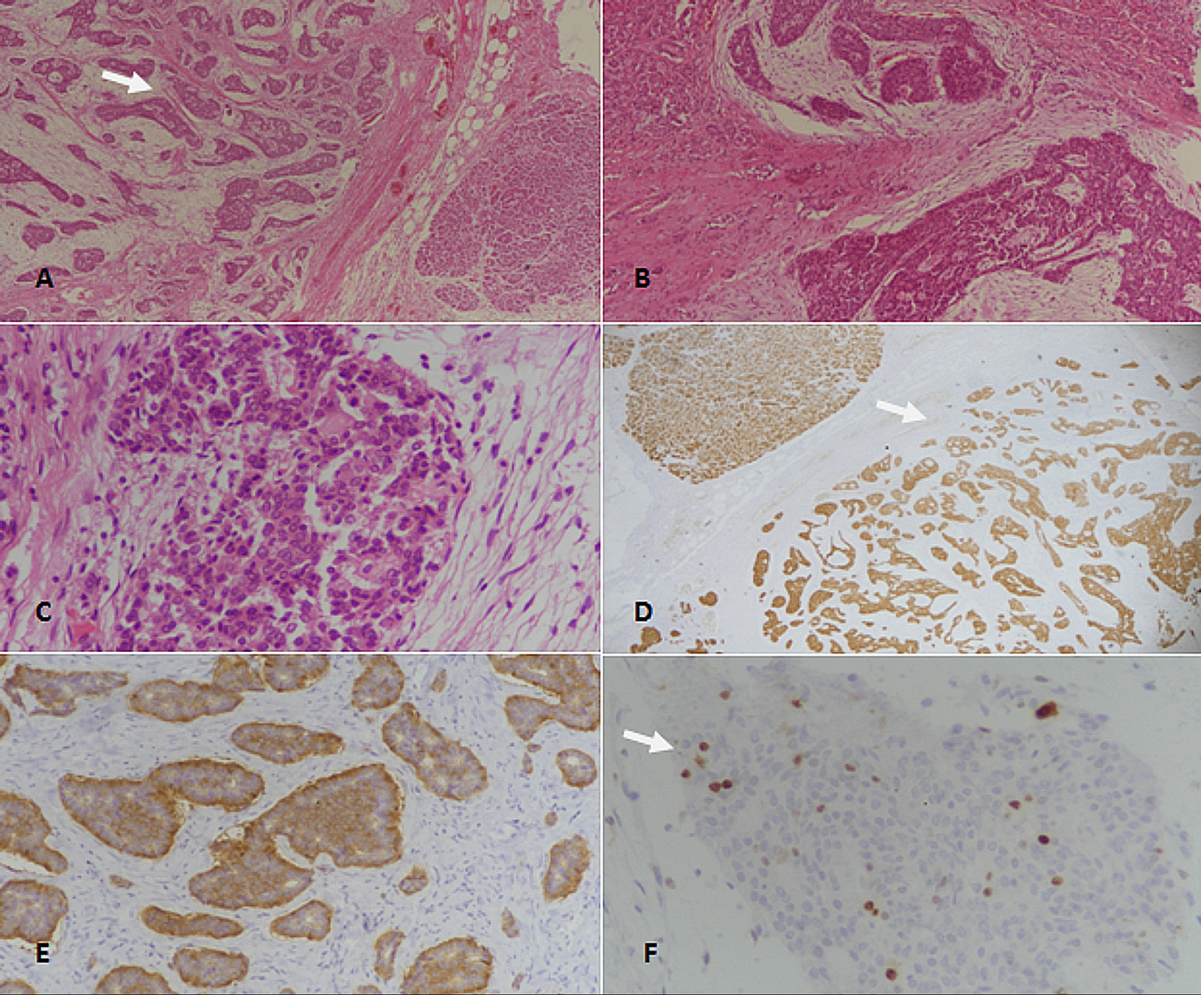
Well-differentiated neuroendocrine tumor (WDNET), grade 2 of the pancreas. (A): H&E-stained section at 40x magnification showing clusters of neoplastic neuroendocrine cells (arrow). (B): H&E-stained section at 100x magnification revealing neuroendocrine tumor with nested architecture. (C): 400x magnification (H&E-staining) showing tumor cells with moderate nuclear atypia and stippled coarse chromatin. (D): Pancytokeratin (CKAE1/AE3)-staining showing diffuse positivity (arrow) in tumor cells. (E): Synaptophysin immunostaining depicting diffuse positivity in tumor cells. (F): Ki67 immunostaining showing 5% proliferative index; nuclear positivity is seen in few tumor cells (arrow). H&E: Hematoxylin and eosin.

**Figure 3:**
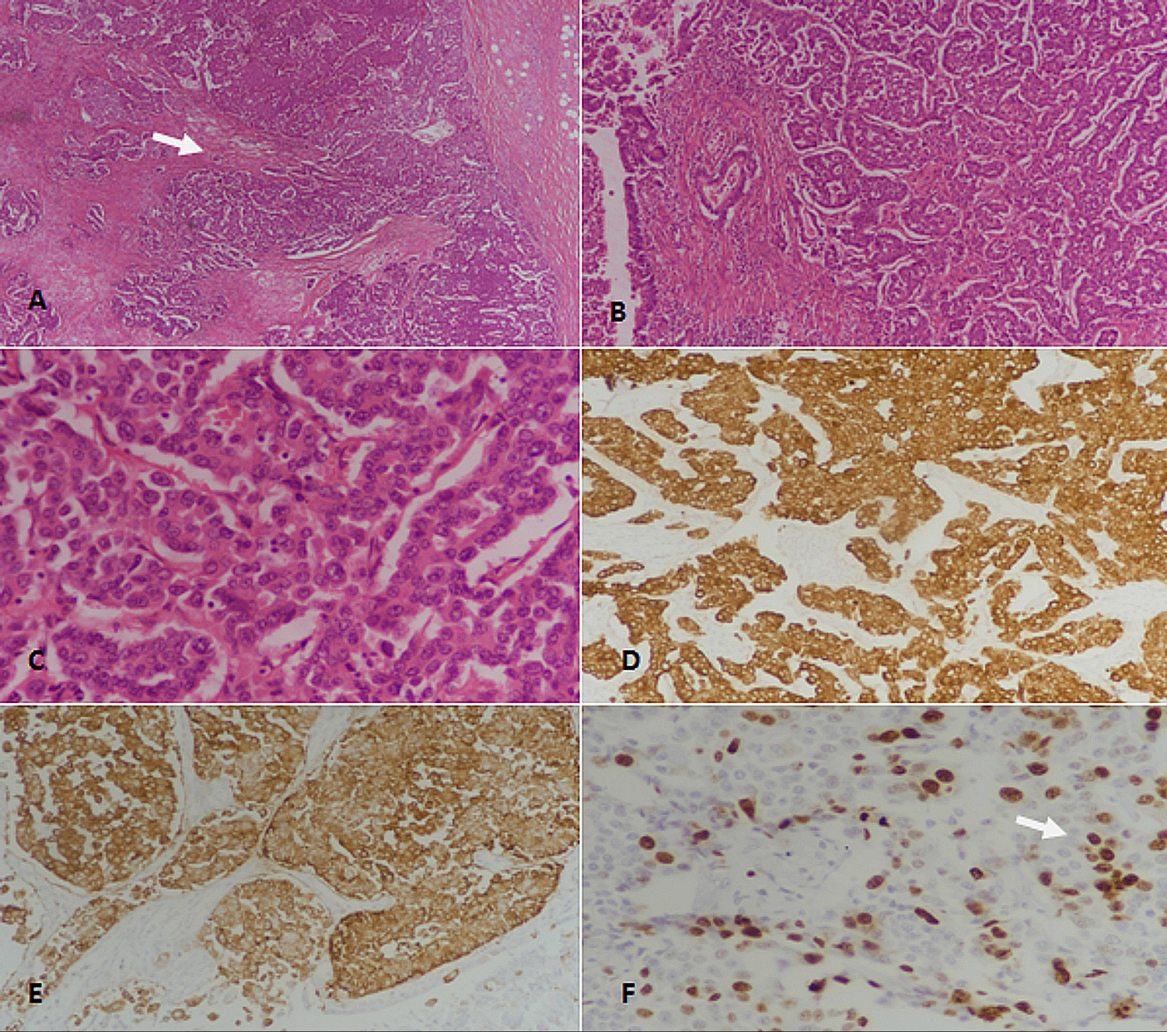
Well-differentiated neuroendocrine tumor (WDNET), grade 3. (A): H&E-stained section at 40x magnification showing nests and trabeculae of tumor cells (arrow). (B): H&E-stained section at 100x magnification revealing neuroendocrine tumor with organoid architecture. (C): 400x magnification (H&E-staining) showing tumor cells with moderate to marked nuclear atypia and coarse chromatin. (D): Pancytokeratin (CKAE1/AE3)-staining showing strong diffuse positivity. (E): Synaptophysin immunostaining depicting diffuse positivity. (F): Ki67 immunostaining showing more than 20% proliferative index; some tumor cells show nuclear positivity (arrow). H&E: Hematoxylin and eosin.

WDNETS were graded numerically into G1 (low), G2 (intermediate), and G3 (high) grades on the basis of the number of mitoses per 2 mm^2^ and Ki67 proliferative index [6]. G1 tumors had less than two mitoses per 2 mm^2^ and a Ki67 index of less than 3%. G2 tumors had 2-20 mitoses per 2 mm^2^ and a Ki67 index between 3% and 20%. G3 tumors had more than 20 mitoses per 2 mm^2^ and a Ki67 index of more than 20% (Figures 1-3).

**Figure 4:**
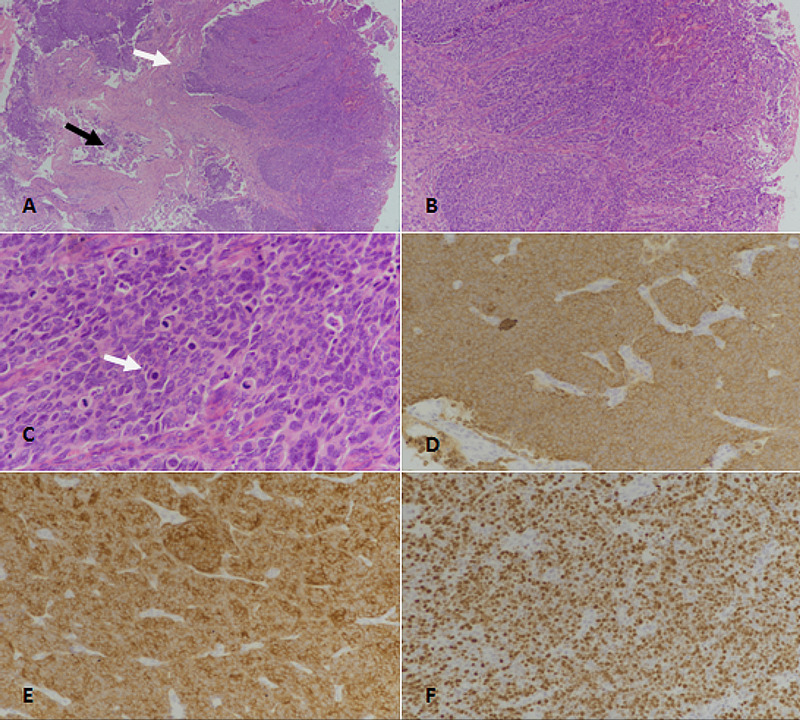
Poorly differentiated neuroendocrine carcinoma (PDNEC). (A): H&E-stained section at 40x magnification showing sheets of tumor cells (white arrow). Areas of necrosis are also evident (black arrow). (B): H&E-stained section at 100x magnification revealing neuroendocrine carcinoma with sheet-like architecture. (C): 400x magnification (H&E-staining) showing tumor cells with marked nuclear atypia and frequent mitoses (white arrow). (D): Pancytokeratin (CKAE1/AE3)-staining showing strong diffuse positivity. (E): Synaptophysin immunostaining depicting diffuse positivity. (F): Ki67 immunostaining showing approximately 80% proliferative index. H&E: Hematoxylin and eosin.

PDNECs were characterized by a sheet-like architecture. Cytologically, the cells are more atypical than WDNETs (Figure 4).

Data analysis was performed using Statistical Package for Social Sciences (Version 26.0, IBM Inc., Armonk, USA). Chi-square, Fisher exact test, and independent t-test were used to check the association. P values ≤ 0.05 were considered significant.

## Results

Our results showed that the mean age of patients was 46.8 ± 17.1 years, and 62.3% belonged to male gender. Majority of the NENs were GI tract origin (86.9%). The most common site of tumor in gastroenteropancreatic tract was the small bowel (31.1%), followed by the stomach (26.2%); 95.1% of the tumors were WDNETs, out of which most common grade was G2. Six cases were of PDNEC, out of which two were SCNEC and four were LCNEC. The mean Ki67 index was 15.8 ± 23.8 (Table 1).

**Table 1:** Clinicopathological features of population under study *Mean ± SD (standard deviation). WDNET, Well-differentiated neuroendocrine tumor; PDNEC, poorly differentiated neuroendocrine carcinoma.

Clinicopathological characteristics	Frequency (%)
Gender	
Male	76 (62.3)
Female	46 (37.7)
Age (years)*	46.8 ± 17.1
Age groups	
<30 years	22 (18)
30–50 years	50 (41)
>50 years	50 (41)
Ki67 index (%)*	15.8 ± 23.8
Tumor size (n = 24)*	2.3 ± 1.5
Tumor size groups	
≤2 cm	10 (41.7)
2.1–4.0 cm	12 (50)
>4 cm	2 (8.3)
Tumor site category	
Gastrointestinal tract	106 (86.9)
Pancreatobiliary tract	16 (13.1)
Individual tumor sites	
Rectum	12 (9.8)
Stomach	32 (26.2)
Small bowel	38 (31.1)
Appendix	12 (9.8)
Anal canal	6 (4.9)
Colon	6 (4.9)
Pancreas	14 (11.5)
Gall bladder	2 (1.6)
Tumor grade	
Grade 1	44 (36.1)
Grade 2	58 (47.5)
Grade 3	20 (16.4)
Tumor (T) stage (n = 24)	
T1	8 (33.3)
T2	0 (0)
T3	12 (50)
T4	4 (16.7)
Type of neuroendocrine neoplasm	
WDNET	116 (95.1)
PDNEC	6 (4.9)

Table 2 compares the features of WDNETs and PDNECs. PDNECs were noted to have a significantly older mean age than WDNETs. No significant difference was noted in terms of the site of origin or gender.

**Table 2:** Association of clinicopathological characteristics with the type of neuroendocrine neoplasm *Fisher’s exact test was applied. **Mean ± SD (standard deviation), independent t-test was applied. ***p value significant as <0.05. WDNET, Well-differentiated neuroendocrine tumor; PDNEC, poorly differentiated neuroendocrine carcinoma.

Clinicopathological characteristics	Frequency (%)		p value
	WDNET	PDNEC	
	(n = 116)	(n = 6)	
Gender*			0.197
Male	74 (63.8)	2 (33.3)	
Female	42 (36.2)	4 (66.7)	
Age (years)**	46.1 ± 16.9	61.7 ± 14.4	0.029***
Age groups*			
<30 years	22 (19)	0 (0)	0.558
31–50 years	48 (41.4)	2 (33.3)	
>50 years	46 (39.7)	4 (66.7)	
Ki67 (%)**	12.6 ± 19.7	76.7 ± 10.3	<0.0001***
Tumor site*			
Gastrointestinal tract	102 (87.9)	4 (66.7)	0.177
Pancreatobiliary tract	14 (12.1)	2 (33.3)	
Individual tumor sites*			
Rectum	10 (8.6)	2 (33.3)	0.217
Stomach	32 (27.6)	0 (0)	
Small bowel	36 (31)	2 (33.3)	
Appendix	12 (10.3)	0 (0)	
Anal canal	6 (5.2)	0 (0)	
Colon	6 (5.2)	0 (0)	
Pancreas	12 (10.3)	2 (33.3)	
Gall bladder	2 (1.7)	0 (0)	

Table 3 shows the association of clinicopathological features of WDNETs with tumor grade. There was a significant difference in terms of age. Grade 3 WDNETs were noted to have an older mean age than grades 1 and 2 WDNETs. Ten out of 102 (9.8%) WDNETs of GI tract were grade 3, compared with four out of 14 (28.5%) of pancreatobiliary tract. Grade 2 and 3 WDNETs had a larger tumor size than grade 1 tumors. Grade 3 WDNETs also had a higher tumor (T) stage than grade 1 and 2 tumors; however, the results were not statistically significant.

**Table 3:** Association of clinicopathological characteristics of well-differentiated neuroendocrine tumors with the tumor grade (n = 116) *Chi-square test was applied. **Mean ± SD (standard deviation), ANOVA was applied. ***Fisher’s exact test was applied. ****p value significant as <0.05.

Clinicopathological characteristics	Frequency (%)			p value
	Grade 1 (n = 44)	Grade 2 (n = 58)	Grade 3 (n = 14)	
Gender*				
Male	30 (68.2)	34 (58.6)	10 (71.4)	0.499
Female	14 (31.8)	24 (41.4)	4 (28.6)	
Age (years)**	40.5 ± 16.2	48.6 ± 15.9	53.3 ± 19.3	0.013****
Age groups***				
<30 years	12 (27.3)	8 (13.8)	2 (14.3)	0.306
31­–50 years	18 (40.9)	26 (44.8)	4 (28.6)	
>50 years	14 (31.8)	24 (41.4)	8 (57.1)	
Ki67 index (%)**	3.1 ± 1.2	8.2 ± 4.9	60.7 ± 21.4	<0.0001****
Tumor size (n = 24)**	1.4 ± 1.0	3.50 ± 1.1	3.50 ± 0.0	<0.0001****
Tumor size groups*** (n = 24)				
≤2 cm	10 (71.4)	0 (0)	0 (0)	0.001****
2.1–4.0 cm	4 (28.6)	6 (75)	2 (50)	
>4 cm	0 (0)	2 (25)	2 (50)	
Tumor site***				
Gastrointestinal tract	42 (95.5)	50 (86.2)	10 (71.4)	0.040****
Pancreatobiliary tract	2 (4.5)	8 (13.8)	4 (28.6)	
Individual tumor sites***				
Rectum	2 (4.5)	6 (10.3)	2 (14.3)	<0.0001****
Stomach	6 (13.6)	24 (41.4)	2 (14.3)	
Small bowel	14 (31.8)	16 (27.6)	6 (42.8)	
Appendix	10 (22.7)	2 (3.4)	0 (0)	
Anal canal	6 (13.6)	0 (0)	0 (0)	
Colon	4 (9.1)	2 (3.4)	0 (0)	
Pancreas	0 (0)	8 (13.8)	4 (28.6)	
Gall bladder	2 (4.5)	0 (0)	0 (0)	
Tumor (T) stage (n = 24)***				
T1	6 (42.9)	2 (25)	0 (0)	0.052
T2	0 (0)	0 (0)	0 (0)	
T3	6 (42.9)	6 (75)	12 (85.7)	
T4	2 (14.3)	0 (0)	2 (14.3)	

Table 4 shows the comparison between GI and pancreatobiliary tract tumors. Pancreatobiliary tract NENs had a higher mean Ki67 index, but the difference was not statistically significant. Pancreatobiliary tract tumors had a significantly higher frequency of grade 3 than GI NETs.

**Table 4:** Association of clinicopathological characteristics with the tumor site *Chi-square test was applied. **Mean ± SD (standard deviation), independent t-test was applied. ***Fisher’s exact test was applied. ****p value significant as <0.05. WDNET, Well-differentiated neuroendocrine tumor; PDNEC, poorly differentiated neuroendocrine carcinoma.

Clinicopathological characteristics	Frequency (%)		p value
	Gastrointestinal tract (n = 106)	Pancreatobiliary tract (n = 16)	
Gender*			
Male	64 (60.4)	12 (75)	0.261
Female	42 (39.6)	4 (25)	
Age (years)**	46.5 ± 17.50	49.4 ± 14.7	0.527
Age groups***			
<30 years	20 (18.9)	2 (12.5)	0.825
31–50 years	44 (41.5)	6 (37.5)	
>50 years	42 (39.6)	8 (50)	
Ki67 index (%)**	13.3 ± 20.2	32.4 ± 37.2	0.061
Tumor grade***			
Grade 1	42 (39.6)	2 (12.5)	0.021****
Grade 2	50 (47.2)	8 (50)	
Grade 3	14 (13.2)	6 (37.5)	
Type of neuroendocrine neoplasm***			
WDNET	102 (96.2)	14 (87.5)	0.177
PDNEC	4 (3.8)	2 (12.5)	

Table 5 compares the clinicopathological features of grade 3 WDNETs with PDNECs. Although PDNECs had higher mean age and Ki67 index, the results were not statistically significant. 

**Table 5:** Comparison of grade 3 well-differentiated neuroendocrine tumors and poorly differentiated neuroendocrine carcinomas with respect to clinicopathological characteristics *Fisher’s exact test was applied. **Mean ± SD (standard deviation), independent t-test was applied. WDNET, Well-differentiated neuroendocrine tumor; PDNEC, poorly differentiated neuroendocrine carcinoma.

Clinicopathological characteristics	Frequency (%)		p value
	Grade 3 WDNET (n = 14)	PDNEC (n = 6)	
			Gender*			
Male	10 (71.4)	2 (33.3)	0.161
Female	4 (28.6)	4 (66.7)	
Age (years)**	53.3 ± 19.3	61.7 ± 14.4	0.354
Age groups*			
<30 years	2 (14.3)	0 (0)	1
31–50 years	4 (28.6)	2 (33.3)	
>50 years	8 (57.1)	4 (66.7)	
Ki67 (%)**	60.7 ± 21.4	76.7 ± 10.3	0.102
Tumor site*			
Gastrointestinal tract	10 (71.4)	4 (66.7)	1
Pancreatobiliary tract	4 (28.6)	2 (33.3)	
Individual tumor sites*			
Rectum	2 (14.3)	2 (33.3)	0.757
Stomach	2 (14.3)	0 (0)	
Small bowel	6 (42.9)	2 (33.3)	
Pancreas	4 (28.6)	2 (33.3)	

## Discussion

In our study, we studied the clinicopathological features of primary neuroendocrine tumors of GI/pancreatobiliary tract with emphasis on high-grade WDNETs. WDNETs were far more frequent than PDNECs, especially in GI tract. We found that the small bowel was the most common site for primary WDNETs. We also noted that grade 3 WDNETs were significantly more common in the pancreatobiliary tract than GI tract and were associated with older mean age. We also noted that PDNECs had a higher proliferative index and mean age than grade 3 WDNETs; the finding was not statistically significant owing to the small number of cases in the PDNEC category.

The WDNETs of the digestive tract were previously classified as grade 1 and grade 2 tumors with a criterion of mitoses and Ki67 index being <2 mitoses/2 mm^2^ and <3% Ki67 index for G1 NETs as well as 2-20 mitoses/2 mm^2^ and 3%-20% Ki67 index for G2 NETs. Tumors that exceeded the mitotic cutoff of more than 20 mitosis/2 mm^2^ or Ki67 index of more than 20% were classified as NECs. The recent amendment introduced WDNET grade 3 into the classification. These tumors retain the morphological features of WDNETs, i.e., organoid histological patterns with nests, cords, trabeculae, ribbons, and rosette formation; however, these tumors have more than 20 mitoses/2 mm^2^ or Ki67 index of more than 20%. The need for this introduction was based on the differences in the pathogenesis of WDNET and PDNEC that impacted the treatment course and clinical outcomes [7]. Moreover, many studies have shown that differentiation status is the most important prognostic factor in determining the clinical course of NENs regardless of their primary site or stage [8,9]. Ishido et al. showed that small bowel tumors that were less than 1 cm had a risk of muscularis propria and lymphovascular invasion along with lymph node metastasis [10].

Grade 3 WDNETs are considered prognostically worse than PDNECs. A study revealed that high-grade component was noted in 48% of WDNETs of GI/pancreatobiliary tract with a median disease-free survival (DFS) of 55 months that was significantly better than PDNECs (median DFS = 11 months) [7]. Similarly, another study revealed 12.9 months overall survival for GI/pancreatobiliary tract PDNECs [11]. Therefore, it is essential to distinguish grade 3 WDNETs from PDNECs.

Immunohistochemical studies are useful in determining the site of origin of NENs. While CK7 and CK20 stains are of limited use in this context, TTF1 and CDX2 are helpful in differentiating lung-origin NENs from GI-origin NENs.

To date, few studies have compared the clinicopathological features of high-grade (grade 3) WDNETs from low-grade (1-2) NETs. While the sample size of our study was small and we compared the results retrospectively, we found significant associations of grade 3 WDNETs in terms of age and location. However, follow-up data were not available; therefore, we could not compare the survival or disease recurrence between different grades of WDNETs. Therefore, based on our results, large-scale prospective trials are warranted to validate our findings and to further reveal the differences between these tumors in terms of recurrence and survival.

## Conclusions

NENs are heterogeneous in terms of disease origin and pathogenesis. Grade 3 WDNETs are recently described. In our study, we noted that grade 3 WDNETs were more common in the pancreatobiliary tract than GI tract. Moreover, grade 3 WDNETs were associated with older mean age than low-grade WDNETs. Further studies are needed to explore more differences between WDNETs of different grades in terms of survival and disease recurrences in our population. In addition, biological differences between WDNETs of different grades should be explored for better patient management. For a pathological perspective, it is extremely important to understand the morphological differences between high-grade WDNETs and PDNECs, as some recent studies have shown a significant difference in the survival between these two groups of NENs, despite comparable proliferative index.

## References

[1] Modlin IM, Kidd M, Latich I, Zikusoka MN, Shapiro MD (2005). Current status of gastrointestinal carcinoids. Gastroenterology.

[2] Hashmi AA, Ali J, Khan K (2020). Clinicopathological spectrum of primary and metastatic neuroendocrine neoplasms. Cureus.

[3] Cives M, Strosberg JR (2018). Gastroenteropancreatic neuroendocrine tumors. CA Cancer J Clin.

[4] Moertel CG (1987). Karnofsky memorial lecture. An odyssey in the land of small tumors. J Clin Oncol.

[5] Yao JC, Hassan M, Phan A (2008). One hundred years after "carcinoid": epidemiology of and prognostic factors for neuroendocrine tumors in 35,825 cases in the United States. J Clin Oncol.

[6] Klimstra DS, Modlin IR, Coppola D, Lloyd RV, Suster S (2010). The pathologic classification of neuroendocrine tumors: a review of nomenclature, grading, and staging systems. Pancreas.

[7] Tang LH, Untch BR, Reidy DL (2016). Well-differentiated neuroendocrine tumors with a morphologically apparent high-grade component: a pathway distinct from poorly differentiated neuroendocrine carcinomas. Clin Cancer Res.

[8] Lepage C, Bouvier AM, Phelip JM, Hatem C, Vernet C, Faivre J (2004). Incidence and management of malignant digestive endocrine tumours in a well defined French population. Gut.

[9] Rindi G, Klöppel G, Alhman H (2006). TNM staging of foregut (neuro)endocrine tumors: a consensus proposal including a grading system. Virchows Arch.

[10] Ishido K, Tanabe S, Higuchi K (2010). Clinicopathological evaluation of duodenal well-differentiated endocrine tumors. World J Gastroenterol.

[11] Milione M, Maisonneuve P, Spada F (2017). The clinicopathologic heterogeneity of grade 3 gastroenteropancreatic neuroendocrine neoplasms: morphological differentiation and proliferation identify different prognostic categories. Neuroendocrinology.

